# The Prevalence of Selected Potential Drug-Drug Interactions of Analgesic Drugs and Possible Methods of Preventing Them: Lessons Learned From the Analysis of the Real-World National Database of 38 Million Citizens of Poland

**DOI:** 10.3389/fphar.2020.607852

**Published:** 2021-01-18

**Authors:** Przemysław Kardas, Filip Urbański, Aneta Lichwierowicz, Ewa Chudzyńska, Marcin Czech, Katarzyna Makowska, Grzegorz Kardas

**Affiliations:** ^1^ Department of Family Medicine, Medical University of Lodz, Łódź, Poland; ^2^ National Health Fund, Warsaw, Poland; ^3^ Department of Pharmacoeconomics, Institute of Mother and Child, Warsaw, Poland; ^4^ Department of Internal Diseases, Asthma and Allergy, Medical University of Lodz, Łódź, Poland

**Keywords:** drug – drug interactions, drug safety, inapropriate prescribing, pharmacoepidaemiology, real-world data, claim database, analgesic drugs, Poland

## Abstract

**Introduction:** Drug-drug interactions may lead to poor health outcomes, as well as increased costs and utilization of healthcare services. Unfortunately, real-world data continuously prove high prevalence of potential drug-drug interactions (pDDIs) worldwide. Among identified drivers, ageing, multimorbidity and polypharmacy play a very important role. With these factors being widespread, the need for implementation of strategies minimizing the burden of pDDIs becomes an urgency. This, however, requires a better understanding of the prevalence of pDDIs and the underlying causative factors.

**Aim of study:** To assess the real-world prevalence of pDDIs and its characteristics in the general population of Poland, using analgesic drugs as a model, and to find out whether pDDIs are caused by prescribing coming from the very same prescribers (co-prescribing).

**Methods:** A retrospective analysis of the 2018 dispensation data of the National Health Fund (NHF) - the only Polish public healthcare payer organization with nationwide coverage. We searched for selected pDDIs of non-steroidal anti-inflammatory drugs (NSAIDs) with antihypertensives, other NSAIDs (double use), oral glucocorticoids, oral anticoagulants, selective serotonin reuptake inhibitors (SSRIs), serotonin–norepinephrine reuptake inhibitors (SNRIs), and antiplatelet drugs; as well as opioides with SSRIs, SNRIs, gabapentinoids, and benzodiazepines. A pDDI was deemed present if two drugs standing in a possible conflict were dispensed within the same calendar month.

**Results:** Out of 38.4 million citizens of Poland, 23.3 million were dispensed prescribed drugs reimbursed by NHF in 2018. In this cohort, we have identified 2,485,787 cases of analgesic drug pDDIs, corresponding with 6.47% of the Polish population. Out of these, the most prevalent pDDI was caused by “NSAIDs + antihypertensives” (1,583,575 cases, i.e., 4.12% of the Polish population), followed by “NSAIDs + NSAIDs” (538,640, 1.40%) and “NSAIDs + glucocorticoids” (213,504, 0.56%). The most persistent pDDIs among those studied were caused by “Opioids + Gabapentinoids” (2.19, 95%CI: 2.16–2.22 months). On average, 76.63% of all cases of pDDIs were caused by drugs prescribed by the very same prescribers.

**Conclusion:** Based on high-quality, nationwide data, we have found a high prevalence of analgesic drugs-related pDDIs in Poland. Over ¾ of the identified pDDIs were caused by co-prescribing, i.e., prescriptions issued by the same prescribers. The significance of the problem, illustrated with our findings on analgesic drugs-related pDDIs in Poland, deserves much more scientific and policymaker attention.

## Introduction

With numerous potent drugs available these days, effective pharmacotherapy became accessible even in cases that were obligatorily treated with other methods in the past. As a consequence, pharmacotherapy is a choice of preference for numerous conditions, and various drugs are almost automatically prescribed by healthcare professionals across wide spectrum of diseases, from mild to those life-threatening ones.

However, this approach has serious shortcomings. The willing use of pharmacotherapy often leads to concurrent usage of multiple drugs in a patient. This, in turn, opens up an avenue for drug-drug interactions (DDIs). DDIs can be defined with a scenario in which the pharmacological or clinical response to the administration of a combination of two drugs is different than expected, based on both drugs known effects when individually prescribed (Mino-León et al., 2011). Not all DDIs are negative or have serious health consequences. However, some of them may lead to loss of treatment effectiveness, adverse drug reactions (ADRs) or toxicity. These may result in clinical manifestations such as failure to achieve treatment goals, deterioration of the patient’s status, or even death (Cascorbi, 2012; Palleria et al., 2013). Moreover, they have a major impact on healthcare services utilization, in particular on hospitalization rates (Ogawa and Echizen, 2010; Palleria et al., 2013). Finally, they have profound economic consequences, leading to an increase in costs incurred by both individuals, as well as healthcare systems (Moore et al., 1998; Bordet et al., 2001; Abdulah et al., 2017; Bethi et al., 2018).

On the other hand, in daily clinical practice, it is quite common to use drug combinations with possible capability to interact. It is undoubtedly the unavoidable consequence of navigating between two opposite directions of maximizing effectiveness of a therapy, and maximizing its safety. Consequently, along with actual DDIs, the concept of potential DDIs (pDDIs) has emerged. It is defined as DDIs that may occur (Mousavi and Ghanbari, 2017).

A number of pDDIs has already been identified and classified. Thus, most of the pDDIs are preventable since they can be predicted based on well-known pharmacological properties of the drugs involved (Ahmad et al., 2015). Unfortunately, despite multiple initiatives to reduce the number of drug interactions undertaken locally or nationally, real-world data continuously prove high prevalence of pDDI worldwide (Mousavi and Ghanbari, 2017).

There are various factors that may significantly increase of the risk of drug-drug interactions. With their growing prevalence, pDDIs more and more often occur as a consequence of interlinked factors of ageing, multimorbidity (concurrent existence of two or more conditions), and polypharmacy (lacking standard definition; it is most often defined as concurrent use of five or more drugs (Mousavi and Ghanbari, 2017)).

Another important cluster of factors is connected with the healthcare system architecture and functioning. Fragmented healthcare, with numerous professionals taking care of the same individual patient and lack of effective follow-up, play an important role in this scenario (Masnoon et al., 2017; Ong et al., 2017; Vehko et al., 2018). The risk of pDDIs is even increased with a lack of or poor communication and data exchange between various healthcare providers (Ong et al., 2017; Vehko et al., 2018). Clinicians also blame insufficient clinical guidance for the management of complex patients, which often leads to inappropriate polypharmacy with increased risk of pDDIs (Cadogan et al., 2016; Masnoon et al., 2017). Another significant problem is poor awareness of deprescribing, that is the possibility of reducing the number of drugs a patient is prescribed (Duncan et al., 2017; Turner and Tannenbaum, 2017). Finally, pDDIs are sometimes triggered by mechanisms that paradoxically encourage overprescribing, or even incentivize it, such as certain contractual arrangements, or external pressures (e.g., exerted by pharmaceutical companies) (Lo et al., 2009).

For all these reasons, various pDDIs can be identified in each and every area of pharmacotherapy, and create a real problem for both individual patients and healthcare systems. Therefore, there is a need for implementation of relevant preventive and corrective measures in order to minimize the negative consequences of DDIs. To maximize the effectiveness of such a solution, it needs to be tailored to specific scenarios. Therefore, a better understanding of pDDIs prevalence and the underlying causative factors is of utmost importance.

This need is particularly clear in Poland - a country with generally high use of drugs (Sharifi et al., 2014). This sort of drug use culture easily leads to polypharmacy. Indeed, current real-world data prove that the prevalence of polypharmacy in Poland is greater than in a number of other European countries (Midão et al., 2018). Assuming that the Polish population is currently one of the oldest ones in Europe and it is still ageing fast (Leszko et al., 2015), the prevalence of polypharmacy in Poland may be expected to continuously rise in the upcoming years. All these conditions provide fertile ground for pDDIs in Poland. Finally, only recently Poland has started the process of digitization of its national healthcare system (Bukowskiand Pogorzelczyk, 2019). Therefore, the basis for the development of the most effective, real-world data-based nationwide mechanisms to mitigate the occurrence of pDDIs has not fully developed yet.

To illustrate the significance of the problem created by pDDIs in Poland, we have adopted for our study a model class of analgesic drugs. These medicines are frequently prescribed for various indications and they are one of the most commonly used groups of drugs (Choudhury and Bezbaruah, 2016). At the same time, they are known to cause a lot of potential drug-drug interactions (Moore et al., 2015; Khandeparkar and Rataboli, 2017). This study focused on clinically significant pDDIs of two or more analgesic drugs as well as those resulting from the concurrent use of analgesics with other drugs. For example, using two or more non-steroidal anti-inflammatory drugs (NSAIDs) at the same time greatly increases the risk of upper digestive tract bleeding (O’mahony et al., 2015).

In the light of the above, the aim of this study was twofold. First of all, we wanted to assess the real-world prevalence of selected potential drug-drug interactions of analgesic drugs and their characteristics in the general population of Poland. In order to lay foundations for future preventive and corrective interventions, we also wanted to find out whether individual clinicians are responsible for prescriptions leading to the risk of pDDIs. Therefore, the secondary aim of this study was to establish whether the identified pDDIs of analgesic drugs were caused by co-prescribing of potentially interacting drugs, i.e., by prescriptions issued by the very same prescribers.

## Methodology

### Data and Study Design

This was a retrospective analysis of the 2018 drugs dispensation data of the Polish National Health Fund (NHF, in Polish: *Narodowy Fundusz Zdrowia*). NHF is the only public healthcare payer in Poland. The NHF’s database possesses nationwide coverage and registers information on dispensation of all reimbursed drugs, regardless of whether a public or private healthcare provider issued a particular prescription.

Within the framework of this study, the NHF database was searched for selected pDDIs of:

(1). non-steroidal anti-inflammatory drugs (NSAIDs) - with selected antihypertensives, NSAIDs (double use), oral glucocorticoids, oral anticoagulants, SSRIs, SNRIs and antiplatelet drugs;
(2). tramadol (big doses of >= 200 mg only) – with SSRI and SNRI;
(3). opioids - with gabapentinoids and benzodiazepines.

A detailed list of potential drug-drug interactions, along with their classification and clinical manifestations justifying selection for this analysis are presented in Table 1. Table 2 specifies the range of particular drugs included in this analysis.

**TABLE 1:** Analgesic drugs pDDIs of interest for this study and their clinical consequences.

pDDI	Category of interaction^a^	Clinical consequence of interaction
NSAIDs + Antihypertensive drugs	C	Increased blood pressure Kalafutova et al. (2014)
NSAIDs + NSAIDs	D	Increased risk of gastrointestinal bleeding and renal failure O’mahony et al. (2015)
NSAIDs + GCs	C	Increased risk of gastrointestinal bleeding Moore et al. (2015)
NSAIDs + SSRI/SNRI	C	Increased risk of gastrointestinal bleeding Perahia et al. (2013)
NSAIDs + OACs	C	Increased risk of gastrointestinal bleeding Battistella et al. (2005)
NSAIDs + Antiplatelet drugs	C	Increased risk of gastrointestinal bleeding Zaremba (2012)
Opioids + Gabapentinoids	C	Increased risk of respiratory depression Gomes et al. (2017)
Opioids + Benzodiazepines	C	Increased risk of respiratory depression Jones and McAninch (2015)
Tramadol + SSRIs/SNRIs	C	Increased risk of seizures, arrhythmia (prolongation of the QT interval) and of serotonin syndrome with high doses of tramadol (≥200 mg) Beakley et al. (2015)

^a^ pDDIs category codes: C, monitor therapy; D, consider therapy modification.

ACEIs, angiotensin-converting enzyme inhibitors; ARBs, angiotensin II receptor blockers; GCs, glucocorticoids; OACs, oral anticoagulants; NSAIDs, non-steroidal anti-inflammatory drugs; pDDI, potential drug-drug interaction; SSRIs, serotonin-selective reuptake inhibitor; SNRIs - serotonin-norepinephrine reuptake inhibitors.

**TABLE 2:** Drug classes and their representatives included in the analysis.

Drug class	Studied drugs
**NSAIDs**	diclofenac, ibuprofen, celecoxib, naproxen, etoricoxib, piroxicam, meloxicam, ketoprofen, dexketoprofen, aceclofenac, lornoxicam, phenylbutazone, mefenamic acid, nimesulide
**Tramadol**	tramadol^a^
**Opioids**	codeine, dihydrocodeine, morphine, oxycodone, buprenorphine, fentanyl
**Benzodiazepines**	alprazolam, diazepam, lorazepam, temazepam, bromazepam
**Gabapentinoids**	gabapentin, pregabalin
**GCs**	prednisone, prednisolone, methylprednisolone
**Antiplatelet drugs**	ticlopidine, clopidogrel, ticagrelor, prasugrel
**Antihypertensive drugs** ^b^	valsartan, losartan, telmisartan, irbesartan, candesartan, enalapril, ramipril, quinapril, perindopril, zofenopril, metoprolol, nebiolol, carvedilol, propranolol, betaxolol
**SSRI/SNRI**	paroxetine, fluoxetine, citalopram, escitalopram/duloxetine, venlafaxine
**OACs**	warfarin, acenocoumarol

^a^ Only big doses of ≥200 mg.

^b^ Anti-hypertensive drugs studied were ACEIs, ARBs, and beta-blockers.

ACEIs, angiotensin-converting enzyme inhibitors; ARBs, angiotensin II receptor blockers; GCs, glucocorticoids; OACs, oral anticoagulants; NSAIDs, non-steroidal anti-inflammatory drugs; SSRIs, serotonin-selective reuptake inhibitors; SNRIs, serotonin-norepinephrine reuptake inhibitors.

It is noteworthy that tramadol, formally belonging to the class of opioids, was analyzed separately. The reason for this was twofold. Firstly, it is the only weak opioid, representing step 2 of the WHO analgesic ladder (Anekar and Cascella, 2020), which is frequently used in Poland. Moreover, it also has a specific formal status in Poland. Unlike typical opioid analgesic drugs, it is not prescribed with special precautions (i.e., it is prescribed on regular prescriptions, rather than special ‘narcotic’ prescriptions as other opioids). The opposite approach is applied for codeine which in Poland has been traditionally classified as a regular opioid, and shares most of formal special precautions with strong opioids. Therefore, in our analysis we grouped it with other opioids.

NSAIDs are known to antagonize the effects of antihypertensive drugs, and these associations can lead to an increase in arterial blood pressure. However, the impact of this pDDI on hypertension control varies depending on an antihypertensive drug class, being most pronounced in the case of angiotensin converting enzyme inhibitors (ACEIs) or angiotensin receptor blockers (ARBs), and practically absent for calcium channel blockers. (Fournier et al., 2012; Kalafutova et al., 2014). Therefore, in our analysis, out of the available antihypertensive drug classes, we included ACEIs, ARBs and beta-blockers only.

### pDDIs Criteria

A pDDI was deemed present if both drugs causing the risk of pDDI for an individual patient were dispensed within the same calendar month. The time interval between the dispensation of the two drugs leading to pDDI was calculated. pDDIs episodes were dichotomized into those being caused by either one or multiple prescribers prescribing drugs standing in possible conflict. For each identified patient with a particular pDDI, the number of calendar months with pDDIs present within the year 2018 was also calculated. Persistence of pDDI was defined with a mean number of months of the year 2018 that a relevant pDDI was present.

For calculation purposes, the national population of Poland in 2018 was assumed to be 38, 413, 139, according to public statistics (Statistical Yearbook of Industry – Poland 2019).

### Ethics

Analyses of aggregated anonymized dispensation data do not involve ethical issues. Therefore, according to the policy of the Ethical Commission of the Medical University of Lodz, these data were not subject to the ethical approval procedure.

### Statistical Analyses

In descriptive statistics, both original numbers and the percentages calculated out of the total number of identified pDDIs were presented, unless otherwise stated. For the analysis of the variance of pDDIs persistence, a Kruskal-Wallis test was used. A *p*-value of less than 0.05 was considered significant.

## Results

Out of 38.4 million citizens of Poland, 23.3 million (60.7%) were dispensed prescribed drugs reimbursed by NHF in 2018. In this cohort, we have identified 2,485,787 cases of analgesic drugs pDDIs of the interest for this analysis, corresponding with 6.47% of the Polish population.

Out of these, the most prevalent pDDIs were caused by the drug pair of “NSAIDs + Antihypertensive drugs” (1,583,575 cases, i.e., 4.12% of the Polish population), followed by the pairs “NSAIDs + NSAIDs” (538,640 cases, i.e., 1.40%) and “NSAIDs + glucocorticoids” (213,504, i.e., 0.56%) (see Table 3).

**TABLE 3:** Prevalence of analgesic drugs pDDIs in Poland in 2018, expressed as number of cases (and corresponding percentages of national population).

pDDI	Number of months with pDDI present^a^												Total
	1	2	3	4	5	6	7	8	9	10	11	12	
NSAIDs + Antihypertensive drugs^b^	992,914 (2.58%)	290,676 (0.76%)	126,927 (0.33%)	69,148 (0.18%)	41,074 (0.11%)	26,243 (0.07%)	13,719 (0.04%)	8,361 (0.02%)	5,784 (0.02%)	4,047 (0.01%)	2,888 (0.01%)	1,794 (0.00%)	**1,583,575 (4.12%)**
NSAIDs + NSAIDs	462,379 (1.2%)	51,461 (0.13%)	13,501 (0.04%)	5,246 (0.01%)	2,639 (0.01%)	1,449 (0.00%)	790 (0.00%)	474 (0.00%)	312 (0.00%)	186 (0.00%)	118 (0.00%)	85 (0.00%)	**538,640 (1.4%)**
NSAIDs + GCs	164,826 (0.43%)	25,039 (0.07%)	10,222 (0.03%)	5,515 (0.01%)	3,292 (0.01%)	2,029 (0.01%)	1,078 (0.00%)	623 (0.00%)	386 (0.00%)	248 (0.00%)	149 (0.00%)	97 (0.00%)	**213,504 (0.56%)**
NSAIDs + SSRI/SNRI	45,951 (0.12%)	9,739 (0.03%)	3,346 (0.01%)	1,637 (0.00%)	893 (0.00%)	486 (0.00%)	244 (0.00%)	135 (0.00%)	95 (0.00%)	72 (0.00%)	50 (0.00%)	18 (0.00%)	**62,666 (0.16%)**
NSAIDs + OACs	34,048 (0.09%)	7,775 (0.02%)	2,799 (0.01%)	1,201 (0.00%)	459 (0.00%)	191 (0.00%)	63 (0.00%)	30 (0.00%)	6 (0.00%)	4 (0.00%)	4 (0.00%)	2 (0.00%)	**46,582 (0.12%)**
NSAIDs + Antiplatelet drugs	15,972 (0.04%)	3,542 (0.01%)	1,418 (0.00%)	740 (0.00%)	381 (0.00%)	253 (0.00%)	135 (0.00%)	88 (0.00%)	42 (0.00%)	30 (0.00%)	16 (0.00%)	12 (0.00%)	**22,629 (0.06%)**
Opioids + Gabapentinoids	9,470 (0.02%)	3,067 (0.01%)	1,553 (0.00%)	1,061 (0.00%)	653 (0.00%)	481 (0.00%)	327 (0.00%)	205 (0.00%)	147 (0.00%)	91 (0.00%)	51 (0.00%)	12 (0.00%)	**17,118 (0.04%)**
Opioids + Benzodiazepines	481 (0.00%)	47 (0.00%)	18 (0.00%)	14 (0.00%)	9 (0.00%)	4 (0.00%)	6 (0.00%)	3 (0.00%)	0 (0.00%)	1 (0.00%)	2 (0.00%)	1 (0.00%)	**586 (0.00%)**
Tramadol + SSRI/SNRI	297 (0.00%)	78 (0.00%)	39 (0.00%)	15 (0.00%)	16 (0.00%)	13 (0.00%)	8 (0.00%)	5 (0.00%)	8 (0.00%)	2 (0.00%)	3 (0.00%)	3 (0.00%)	**487 (0.00%)**
**TOTAL**	**1,726 338 (4.49%)**	**391,424 (1.02%)**	**159,823 (0.42%)**	**84,577 (0.22%)**	**49,416 (0.13%)**	**31,149 (0.08%)**	**16,370 (0.04%)**	**9,924 (0.03%)**	**6,780 (0.02%)**	**4,681 (0.01%)**	**3,281 (0.01%)**	**2,024 (0.01%)**	**2,485,787 (6.47%)**

^a^ Number of calendar months of year 2018 with particular pDDI present; # big doses of >= 200 mg only.

^b^ Anti-hypertensive drugs studied were ACEIs, ARBs and beta-blockers.

ACEIs, angiotensin-converting enzyme inhibitors; ARBs, angiotensin II receptor blockers; GCs, glucocorticoids; OACs, oral anticoagulants; NSAIDs, non-steroidal anti-inflammatory drugs; pDDI, potential drug-drug interaction; SSRIs, serotonin-selective reuptake inhibitor; SNRIs, serotonin-norepinephrine reuptake inhibitors.

Persistence of the studied pDDIs was quite diverse. Over 2/3 of the identified pDDIs (1,726,338 cases, 69.4% of all the pDDI cases) occurred for in 1 month only, whereas, as many as 91.6% (2,277,585 cases) were present for up to 3 months (Table 3). On the other hand, 2,024 cases of analgesic drugs pDDIs (0.1%) were present in each calendar month of the year 2018, most often being caused by the pair “NSAIDs + Antihypertensive drugs” (1,794 cases). What is interesting, however, is that within each of the studied types of pDDIs, there were some cases of patients who, within each calendar month of the year 2018, were prescribed drugs that were in possible conflict.

**FIGURE 1:**
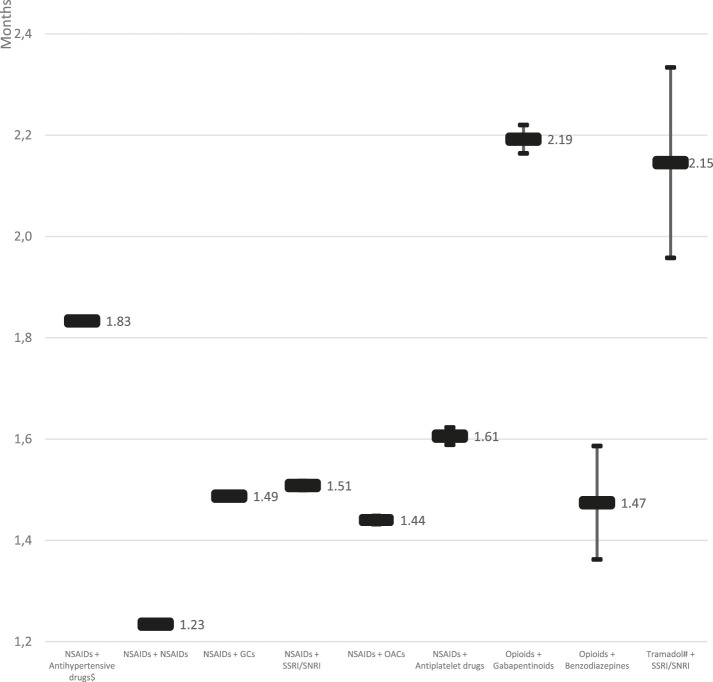
Persistence of pDDIs cases of analgesic drugs, as identified in Polish population in 2018 (means and 95% CI; *p* < 0.001). #big doses of ≥200 mg only; $ anti-hypertensive drugs studied were ACEIs, ARBs and beta-blockers. Note: CI – confidence interval, GCs – glucocorticoids, OACs – oral anticoagulants, NSAIDs – non-steroidal anti-inflammatory drugs, pDDI – potential drug-drug interaction, SSRIs – serotonin-selective reuptake inhibitors, SNRIs – serotonin-norepinephrine reuptake inhibitors.

There was a significant variation (*p* < 0.001) in the persistence across the studied pDDIs. The most persistent out of the analyzed pDDI was caused by “Opioids + Gabapentinoids” (2.19; 95%CI: 2.16–2.22 months), followed by “Tramadol + SSRI/SNRI” (2.15; 95%CI: 1.96–2.33 months), and “NSAIDs + antihypertensives” (1.83; 95%CI: 1.83–1.84 months), whereas the least persistent by “NSAIDs+NSAIDs” (1.23; 95%CI: 1.23–1.24 months) (see Figure 1 for details).

On average, 76.6% of all the cases of pDDIs were caused by prescriptions coming from the very same prescribers, with the high of 87.3% for “Opioids + Gabapentinoids,” and the low of 45.3% for “NSAIDs + SSRI/SNRI” (Table 4). The average percentage of months with pDDIs caused by co-prescribing was even higher and reached the level of 78.7%.

**TABLE 4:** pDDIs resulting from prescriptions coming from the very same prescriber: cases and corresponding patient-months.

pDDI	Number of cases	% Patients receiving Rx leading to pDDI from the same prescriber	Number of patient-months	% Patient-months with pDDIs caused by Rx from the same prescriber
NSAIDs + Antihypertensive drugs	**1 583 575**	**78.3%**	**2 903 210**	**80.2%**
NSAIDs + NSAIDs	**538 640**	**75.4%**	**664 985**	**76.2%**
NSAIDs + GCs	**213 504**	**77.1%**	**317 551**	**79.6%**
NSAIDs + SSRIs/SNRIs	**62 666**	**45.3%**	**94 525**	**49.0%**
NSAIDs + OACs	**46 582**	**73.1%**	**67 083**	**75.5%**
NSAIDs + Antiplatelet drugs	**22 629**	**71.3%**	**36 340**	**74.2%**
Opioids + Gabapentinoids	**17 118**	**87.3%**	**37 525**	**85.7%**
Opioids + Benzodiazepines	**586**	**71.3%**	**864**	**74.4%**
Tramadol^a^ + SSRIs/SNRIs	**487**	**60.2%**	**1 045**	**64.7%**
**TOTAL**	**2 485 787**	—	**4 123 128**	—
**MEAN**	—	**76.6%**	—	**78.7%**

^a^ Only high doses of ≥200 mg, $ anti-hypertensive drugs studied were ACEIs, ARBs and beta-blockers.

ACEIs, angiotensin-converting enzyme inhibitors; ARBs, angiotensin II receptor blockers; GCs, glucocorticoids; OACs, oral anticoagulants; NSAIDs, non-steroidal anti-inflammatory drugs; pDDI, potential drug-drug interaction; Rx – prescriptions; SSRIs, serotonin-selective reuptake inhibitors; SNRIs, serotonin-norepinephrine reuptake inhibitors.

## Discussion

### Drug Interactions – A Global Trend

To the authors’ knowledge, this is the first large, nationwide population-based study on the pDDIs prevalence in Poland. In fact, the sole other Polish study in this area that we have identified in the published literature assessed prevalence of pDDIs in 43 patients of intensive care unit only (Łój et al., 2017). Considering the size of the database used, covering all 38 million of Polish citizens, it is one of the very few such wide-scale studies worldwide. This study provides new, important information on pDDIs. Based on high-quality, nationwide data, we have demonstrated that the prevalence of analgesic drugs-related pDDIs in Poland is high, reaching a number corresponding to 6.47% of the general population. It might be speculated that the reason why this phenomenon occurs is insufficient awareness of the consequences of pDDIs among physicians. Undoubtedly, a lack of availability of nationwide electronic health record system (which is now in a testing phase only) increases the risk of such pDDIs occurrence. The same is true for proprietary databases and clinical decision support systems which are only rarely used in Poland.

It is not easy to directly compare our observations with the results of other studies conducted in various countries due to diverse frameworks used to define pDDIs in terms of study periods varying from one month (Merlo et al., 2001) to several years (Linnarsson, 1993), different definitions of studied cohorts (e.g., outpatients, elderly, polymedicated patients), severity of pDDIs etc. Therefore, prevalence of pDDIs observed in general populations vary significantly ranging from 1.2 to 1.3% in Switzerland (Bucher et al., 2016), 4% in France (Létinier et al., 2019), 8.5% in Italy (Tragni et al., 2013), up to 9.3% in Slovenia (Jazbar et al., 2018). Out of these studies, the last one is particularly interesting. It covered the entire national population of 1.17 million outpatients and focused on clinically relevant pDDIs only (Jazbar et al., 2018).

Reports from outside Europe provide even higher rates of pDDIs. A study assessing outpatients in Taiwan over a period of 3 months reported prevalence of pDDIs as high as 25.6% (Lin et al., 2011). A recent analysis of prescriptions issued to outpatients of a general hospital in China revealed that as many as 30.29% of them contained pDDI with C, D or X risk rating (of which category C stands for ‘monitor therapy,’ D – ‘consider therapy modification,’ and X – ‘avoid combination,’ respectively) (Ren et al., 2020). Finally, among prescriptions filled at a university health center pharmacy in Jamaica, prevalence of pDDIs was 49.8% (of which 4.7% were classified as major, 80.8% as moderate and 14.5% as minor pDDIs) (Kennedy-Dixon et al., 2015).

Undoubtedly, pDDIs come more often with age. In elderly patients attending the public primary health care system in five Brazilian cities, prevalence of pDDIs was high and reached 47.4% of which 33.4% were rated as major by at least 3 of the DDI-checker programs utilized (Neto et al., 2012). On the other hand, pDDIs occur also in pediatric patients. In a sample of Swedish pediatric population aged up to 17 years, 12% of children had at least two dispensed drugs. In this group the prevalence of pDDIs belonging to category C and D was found to be 1.3 and 0.14%, respectively (Holm et al., 2019).

Thus, the results of our analysis possibly reflect a global trend in pharmacotherapy. Therefore, it is of the utmost importance for the national health policy to keep in mind a rising global trend in pDDIs prevalence over time. For example, in a large Scottish database, along with the rise of polypharmacy prevalence, a dramatic increase in potentially serious DDIs was observed, from 5.8% of adults in 1995 having at least one DDI to 13.1% in 2010 (Guthrie et al., 2015). A recent analysis of a French national insurance database clearly proved a rise in pDDIs involving selected analgesic drugs (i.e., NSAIDs and tramadol) between 2006 and 2016 (Souty et al., 2020).

### Potential Drug-Drug Interactions of Analgesic Drugs in Poland

Among the cases of pDDIs identified in our study, we could observe a full spectrum of interactions of clinical importance. On the one hand, the most prevalent (63.7%) were the ones caused by concomitant prescription of NSAIDs and antihypertensive drugs, which might be interpreted as less dangerous. On the other hand, we observed as many as 46,582 cases (i.e., 1.9% of the total number of identified interactions) of pDDIs caused by concomitant prescription of NSAIDs and OACs, which might be regarded as potentially life-threatening (Kent et al., 2018; Carpenter et al., 2019).

High prevalence of analgesic drugs pDDIs in Poland may be at least partly explained by the fragmented healthcare. It still happens very often that patients are managed by dedicated specialists who take care of certain conditions, e.g., musculoskeletal diseases or heart diseases. Poor interprofessional communication in such circumstances may easily result in concomitant prescribing of drugs coming in a potential conflict. Additionally, in Poland some patient-related reasons, e.g., poor health literacy and unhealthy lifestyle, may be especially important. In particular, physical activity is not popular among elderly citizens, which leads to high prevalence of arthropathy and an increased need for painkillers.

Analgesic drugs interactions belong to the most frequent and clinically significant ones. Out of 12 clinically important pDDIs studied in a sample of residents of Regione Emilia-Romagna in Italy, the most prevalent one was caused by the combination of NSAIDs and warfarin (76.7%) (Gagne et al., 2008). In a recent analysis of a French health insurance system database, the prevalence of pDDI in 2016 was estimated at 3.7% for NSAIDs + ARBs/ACEIs, 1.5% for NSAIDs + antiplatelet agents, 0.76% for tramadol + serotonergic drugs and 0.24% for NSAIDs + OACs (Souty et al., 2020). In noncancer pain patients receiving a long-acting opioid, 5.7% were found to be exposed to a potential major drug-drug interaction. What is particularly important is the fact that in this cohort monthly health care costs in a 90-days follow-up period were significantly greater ($3,366 vs. $2,757, a $609 difference) in patients exposed to a potential drug-drug interaction of major clinical significance as compared to those not exposed to a drug-drug interaction (Pergolizzi et al., 2014). Even over-the-counter NSAIDs are known to trigger adverse drug reactions and drug-drug interactions (Moore et al., 2015).

However, it was a very surprising finding of our study that as many as 0.5 million individuals (538,640 cases, i.e., 21.7% of the total number of identified pDDIs) obtained prescriptions for two different NSAIDs. This type of pDDI might be interpreted as a classic case of erroneous prescribing, not adding additional value to the treatment, and seriously rising the risk of ADRs (O’mahony et al., 2015). Besides, this kind of pDDI is very easily identifiable by every prescriber, even without any use of sophisticated software.

Another observation made in our study concerned the persistence of pDDIs. As it is presented in Figure 1, various pDDIs were characterized by persistence periods varying significantly. The most persistent pDDI was caused by “Opioids + Gabapentinoids” (2.19; 95%CI: 2.16–2.22 months), and “Tramadol + SSRI/SNRI” (2.15; 95%CI: 1.96–2.33 months). Although these pDDIs were not that prevalent (17.118 and 487 identified cases, respectively), their clinical significance is very high due to possibly profound consequences. To the contrary, the third in terms of its persistence out of studied pDDIs, i.e., “NSAIDs + antihypertensives” (1.83; 95%CI: 1.83–1.84 months) was the most prevalent of all. Therefore, it should undoubtedly be the target of future preventive interventions.

### Potential Drug-Drug Interactions Coming From Co-prescribing

What is of utmost importance is our finding that on average, over ¾ of the identified pDDIs came from the prescriptions issued by the very same prescribers for individual patients, both in case of pDDIs of lower clinical significance, as well as potentially life-threatening pDDIs (e.g., “NSAIDs + NSAIDs,” 75.4%, “Opioids + Benzodiazepines,” 71.3%, respectively).

High rates of this type of co-prescriptions have also been reported in France (up to 58% of pDDIs, even for pDDI with high clinical relevance (Souty et al., 2020)) and Italy (70.7%) (Tragni et al., 2013).

Obviously, co-prescribing may come from a thoughtful decision and be accompanied by instructed involvement of the patient in the therapy monitoring. Unfortunately, it may be also just a consequence of inadequate pharmacological knowledge of a prescriber. Nevertheless, it should be emphasized that currently in Poland mechanisms alerting clinicians against pDDIs, such as dedicated software, are used very infrequently.

### Clinical and Health Policy Implications

In order to minimize the negative consequences of pDDIs, there is an urgent need to employ various tailored preventive and corrective mechanisms. The results of our study provide important guidance for these initiatives. Our findings prove that at the moment, most of the analgesic drugs pDDIs are generated by the same prescribers, who, for the reasons yet unknown, seem to ignore that fact. This finding has very important clinical implications as most probably the same might be true for pDDIs involving other classes of drugs. Therefore, there is an urgent need for further studies to better understand whether Polish clinicians are aware of the negative consequences of pDDIs. If it is not the case, then dedicated educational activities should be implemented in post-graduate and pre-graduate professional training. Moreover, other studies should be undertaken to establish whether these clinicians are motivated enough to decrease the number of pDDIs, and which strategies could be most effective in solving the problem. Finally, practical support should be provided to those willing to reduce the burden of pDDIs.

So far, a variety of approaches have been adopted to minimize prevalence of pDDIs, and some of them proved to be very efficient. Dedicated educational campaigns targeted at healthcare professionals may increase the awareness of pDDIs, and the methods to prevent them. An example of such an initiative was a successful Italian prescriber-oriented educational campaign which resulted in reduction of the most prevalent DDIs in polymedicated elderly patients, especially NSAIDs-related DDIs (Raschi et al., 2015).

A simple, yet often overlooked, tool in managing DDIs is medication review. This tool can and, in fact, should be often applied by general practitioners and other physicians. Studies show that training in medication review and DDIs raises medication appropriateness (Mahlknecht et al., 2019). Moreover, medication reviews may be also successfully performed by community pharmacists (Vinciguerra et al., 2018; Schindler et al., 2020).

Another promising approach is the widespread use of computerized support systems to alert clinicians about the risk of a pDDI while prescribing. Similar systems may be useful at the community pharmacy level, too. Numerous solutions are commercially available and some of them are free-of-charge (Roblek et al., 2015). An example is Simcyp software, a tool that enables its users to track down DDIs using physiologically-based pharmacokinetic models of CYP450 modulators (Marsousi et al., 2018). However, an important limitation of that approach is a high rate of pDDIs detected (with some of them of minor clinical importance) and sometimes beyond the scope of performance of a clinician. For example, in a study in primary healthcare settings in Turkey, as many as 33% of prescriptions issued for elderly were found to have trigger various pDDIs according to the software used (Gören et al., 2017). Moreover some data show that various software alerts for different pDDIs, with an overlap of final results as low as 11% (Roblek et al., 2015). Thus, as always at the interface of clinical medicine and data-driven decisions, specific precautions are required.

So far, such support systems have not been often used in Poland. Fortunately, the recent implementation of the first modules of a nationwide eHealth system, and particularly the countrywide introduction of e-prescriptions at the beginning of 2020, creates a perfect opportunity to consider such improvements in the upcoming years. With its development and further advancements in digitalization of the healthcare system in Poland, such a solution creates solid grounds for drug interaction management.

In real life conditions, an important question is whether busy clinicians will be able and willing to devote their time to tracing all possible pDDIs. In the light of this question, other approaches fall within the scope of the NHF interest. One of them is pharmaceutical care, a concept that is still in its infancy in Poland. With their specific professional knowledge and general availability, pharmacists possess the capacity to track and manage pDDIs. Along with eHealth systems and specific tools, these professionals may greatly contribute to management of drug interactions and, consequently, to patients’ safety.

A good example of a possible role of pharmacists in prevention of negative effects of polypharmacy, including pDDIs, comes from Scotland. The Scottish Government produced comprehensive guidelines for the prevention and management of polypharmacy (Scottish Government Polypharmacy Model of Care Group Polypharmacy Guidance, 2020) and provided funds for pharmacists to work in general practice to support the delivery of appropriate polypharmacy management (Mair et al., 2019).

### Study Limitations

Certain limitation of this study is lack of the systematic approach toward selection of the pDDIs for the analysis. Unfortunately, no single document or guideline includes a list of clinically relevant pDDis in general, nor analgesic drugs-related pDDIs, in particular. In the case of a lack of such a guidance document, several published studies used proprietary databases for selecting relevant pDDIs. This approach, however, may also lead to marked bias, as there is a substantial discrepancy between pDDIs classification according to the commercially available tools (Schjøtt et al., 2020) and more importantly, between the databases and clinicians’ assessment (Armahizer et al., 2013). Finally, the use of such databases is very limited in Poland. For all these reasons, we decided to look for a wide range of analgesic drugs pDDIs, from less to those more severe ones, which according to their health consequences were deemed clinically important. This large choice of pDDIs was also justified by the pioneer nature of this study, which in fact, was the first of its kind in Poland.

Due to the scope of the data analyzed, the prevalence of the pDDIs found in this study should be accepted as a conservative estimation. At first, the scope of the drugs analyzed was narrowed down to the ones most often prescribed in Poland. These medications, however, do not end up the list of drugs within the classes discussed. Moreover, our operational definition of pDDI was narrowed to dispensation of two drugs coming in a possible conflict within the same calendar month. In fact, even a longer time interval between dispensations may generate pDDI, as the risk of an interaction may come with every single episode of intake of the conflicting drugs. Another confounding factor beyond our observation was patient non-adherence which most often leads to underuse of prescribed drugs, however, less frequently, e.g., in the case of analgesic drugs, may take the opposite direction (Cryer et al., 2016). Out of the studied analgesic drugs, some NSAIDs are available as OTC drugs in Poland. Due to this fact, their dispensation is not registered by the NHF, which make a proportion of pDDIs untraceable within the framework of this study.

Finally, as the study was based on a dispensation database with nationwide coverage (38 million cases), we believe the selection bias of our results is neglectable.

Thus, the significance of the problem of pDDIs, illustrated with our findings on pDDIs for analgesic drugs in Poland is high, and certainly deserves much more scientists and policymakers attention.

## Conclusion

Based on high-quality, nationwide data, this study proved a high prevalence of analgesic drugs-related pDDIs in Poland. Over ¾ of the identified pDDIs were caused by prescriptions issued by the same prescribers, which raises questions regarding the reasons for this scenario. The significance of the problem, illustrated with our findings on analgesic drugs pDDIs in Poland, deserves much more scientific and policymaker attention. Various approaches, from educational campaigns to the use of computerized support systems could be suggested as possible methods to overcome the problem.

## Data Availability Statement

The data analyzed in this study is subject to the following licenses/restrictions: The data that support the findings of this study are available from NHF (data owner). Restrictions apply to the availability of these data, which were used under license for this study. Data are available from the authors with the permission of NHF. Requests to access these datasets should be directed to pkardas@csk.am.lodz.pl.

## Author Contributions

Study concept and design: AL, EC, FU, GK, KM, MC, and PK. Analysis: AL, GK, and PK. All authors participated in the interpretation of the results, drafting and reviewing the manuscript, and approved the final version.

## Conflict of Interest

PK received speaker’s honoraria from Aflofarm, Fresenius, Polpharma and Sandoz; and got funding from a grant from European Union’s Horizon 2020 for GATEKEEPER project (grant agreement N° 857223), and The European Commission ERASMUS+ Project Skills4Adherence (Grant Agreement Number: 2017-1-PL01-KA202-038672), outside this work.

The remaining authors declare that the research was conducted in the absence of any commercial or financial relationships that could be construed as a potential conflict of interest.
